# An Update on the Molecular and Cellular Basis of Pharmacotherapy in Type 2 Diabetes Mellitus

**DOI:** 10.3390/ijms24119328

**Published:** 2023-05-26

**Authors:** Mohamed Omer Mahgoub, Ifrah Ismail Ali, Jennifer O. Adeghate, Kornélia Tekes, Huba Kalász, Ernest A. Adeghate

**Affiliations:** 1 Department of Anatomy, College of Medicine & Health Sciences, United Arab Emirates University, Al Ain P.O. Box 17666, United Arab Emirates; 2 Department of Health and Medical Sciences, Khawarizmi International College, Abu Dhabi P.O. Box 25669, United Arab Emirates; 3 Department of Ophthalmology, Vagelos College of Physicians and Surgeons, Columbia University, 630 W. 168th St., New York, NY 10032, USA; 4 Edward S. Harkness Eye Institute, Columbia University Irving Medical Center, 635 W. 165th St., New York, NY 10032, USA; 5 Department of Pharmacodynamics, Faculty of Pharmacy, Semmelweis University, 1089 Budapest, Hungary; 6 Department of Pharmacology and Pharmacotherapy, Faculty of Medicine, Semmelweis University, 1089 Budapest, Hungary; 7 Zayed Centre for Health Sciences, United Arab Emirates University, Al Ain P.O. Box 15551, United Arab Emirates

**Keywords:** type 2 diabetes mellitus, insulin resistance, diabetes complications, hyperglycemia, physical activity, diet, hypoglycemic agents, lifestyle, treatment, remission of diabetes mellitus

## Abstract

Diabetes mellitus (DM) is a chronic illness with an increasing global prevalence. More than 537 million cases of diabetes were reported worldwide in 2021, and the number is steadily increasing. The worldwide number of people suffering from DM is projected to reach 783 million in 2045. In 2021 alone, more than USD 966 billion was spent on the management of DM. Reduced physical activity due to urbanization is believed to be the major cause of the increase in the incidence of the disease, as it is associated with higher rates of obesity. Diabetes poses a risk for chronic complications such as nephropathy, angiopathy, neuropathy and retinopathy. Hence, the successful management of blood glucose is the cornerstone of DM therapy. The effective management of the hyperglycemia associated with type 2 diabetes includes physical exercise, diet and therapeutic interventions (insulin, biguanides, second generation sulfonylureas, glucagon-like peptide 1 agonists, dipeptidyl-peptidase 4 inhibitors, thiazolidinediones, amylin mimetics, meglitinides, α-glucosidase inhibitors, sodium-glucose cotransporter-2 inhibitors and bile acid sequestrants). The optimal and timely treatment of DM improves the quality of life and reduces the severe burden of the disease for patients. Genetic testing, examining the roles of different genes involved in the pathogenesis of DM, may also help to achieve optimal DM management in the future by reducing the incidence of DM and by enhancing the use of individualized treatment regimens.

## 1. Introduction

Diabetes mellitus (DM) is a chronic disease characterized by various metabolic abnormalities that lead to high blood glucose levels. At present, the International Diabetes Federation (IDF) reports that more than 537 million individuals (aged between 20 and 70 years) worldwide are diabetic, and it is expected that this figure will increase to 783 million by 2045 [1,2]. In 2021, USD 966 billion was spent on managing DM [2]. Multiple factors contribute to the high prevalence of DM such as urbanization and physical inactivity, which leads to increased rates of obesity. Different drugs are known to predispose individuals to DM after prolonged use, such as glucocorticoids, statins, thiazide diuretics, atypical antipsychotics and nucleoside reverse transcriptase inhibitors [3,4].

DM includes type 1, type 2, gestational diabetes and maturity-onset diabetes of the young (MODY). While the different types of DM share common aspects such as elevated blood glucose levels and dyslipidemia, they differ in etiology, clinical manifestations and management [4,5,6,7]. Type 1 DM (T1DM), previously known as insulin-dependent diabetes mellitus, is characterized by the autoimmune destruction of pancreatic beta cells, which are responsible for the production of insulin. As a result, individuals with this type of disease suffer from the deprivation of insulin. On the other hand, type 2 DM (T2DM), which accounts for about 90% of all cases of DM, is characterized by a partial or complete loss of insulin sensitivity in body cells and tissues, a mechanism called peripheral insulin resistance [8,9]. Gestational diabetes (GD) is a temporary condition that is associated with hyperglycemia during pregnancy [10]. It occurs as a result of perturbations in the levels of several hormones, including estrogen, progesterone, growth hormone, cortisol and human placental lactogen, which cause abnormalities in insulin levels and glucose metabolism [11]. Insulin resistance and central obesity can also lead to the development of GD [12]. Indeed, it has been reported that all the markers (insulin, adiponectin and homeostatic model assessment for insulin resistance) of insulin resistance (IR) increase during the period of GD12. This increased IR significantly promotes the association between the waist–hip ratio (WHR) and waist circumference (WC) and GD.

MODY is the rarest type of DM, comprising approximately 1% of cases, and is characterized by mutations in genes that are involved in glucose metabolism. It can often be confused with T1DM and T2DM, although it can be associated with as many complications as the more common types of DM [13,14,15].

## 2. Insulin Resistance

Insulin resistance occurs due to lifestyle factors such as obesity, smoking, physical inactivity and alcohol consumption. These lead to a reduction in insulin sensitivity in the liver, adipose tissues and skeletal muscle, which are the major tissues responsible for glucose uptake and metabolism [16]. 

**Figure 1:**
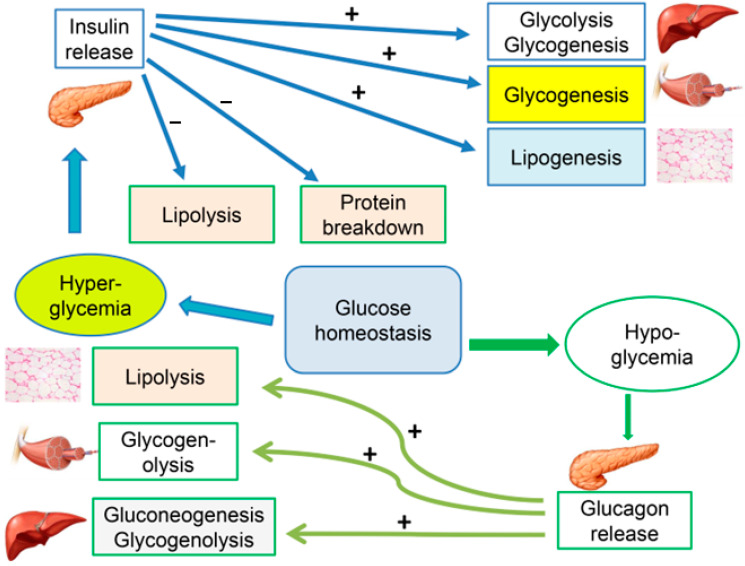
Cellular mechanisms for maintaining glucose homeostasis. Note the interplay between glycolysis, gluconeogenesis, glycogenolysis, glycogenesis, lipolysis and lipogenesis, which take place in the liver, muscle and fat tissues. (+) stimulation; (−) inhibition.

Type 2 DM is a metabolic disorder in which insulin sensitivity is disturbed, affecting its activity in the liver, skeletal muscle and fat cells. Glucose is the main player in insulin metabolism; therefore, maintaining normoglycemia is the main target in the management of T2DM [17]. In order to achieve glucose homeostasis, there are six processes that are tightly regulated both in fed and fasting states. These processes include glycolysis, gluconeogenesis, glycogenolysis, glycogenesis, lipolysis and lipogenesis. In healthy individuals, these processes are finely regulated via the balanced actions of both insulin and glucagon through feedback mechanisms and crosstalk between various organs, including the pancreas, liver, skeletal muscles and adipose tissues (Figure 1) [18].

**Figure 2:**
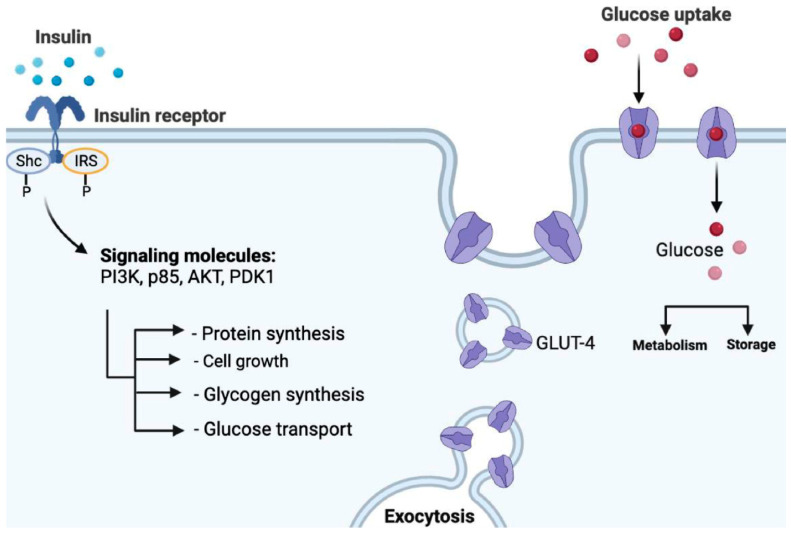
Mechanism of action of insulin: insulin binds with the insulin receptor on the surface of the target cell, leading to the phosphorylation of insulin receptor substrate (IRS) as well as Src homology and collagen protein (Shc). This step is followed by the activation of several signaling molecules (PI3K, p85, AKT and PDK1) which, among others, stimulate the transfer of GLUT4 to the plasma membrane. GLUT4 then assists in the uptake of glucose into the target cell.

After its secretion, insulin binds to the extracellular domain of its receptor, which will cause a series of phosphorylations of different intracellular proteins. This leads to the migration of insulin-responsive glucose transporter 4 (GLUT4) to the plasma membrane and facilitates glucose uptake into the cell [19,20,21,22,23,24,25] (Figure 2).

Different mechanisms have been proposed to explain the molecular events causing insulin resistance. The first mechanism proposes that a reduction in the IRS-1 and IRS-2 substrates involved in insulin signaling is the main cause for reduced insulin activity [22,23,24]. The reduced phosphorylation of these substrates causes the reduced translocation of GLUT4, which interferes with glucose uptake and leads to hyperglycemia. Another proposed theory suggests the involvement of free fatty acids (FFAs) in reducing the sensitivity of insulin due to their deposition in the liver, pancreas and muscle—this occurs as a result of the decreased capacity of the subcutaneous and visceral adipose tissues to store fatty acids, leading to lipotoxicity [26,27]. 

**Figure 3:**
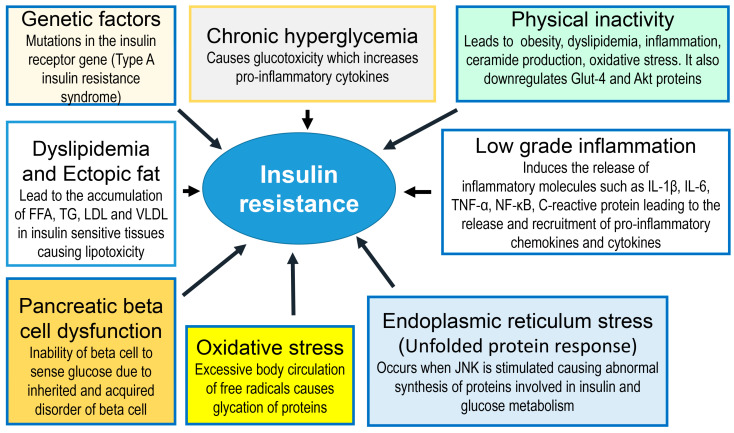
Tissue pathophysiology of type 2 diabetes mellitus. Insulin resistance is caused by a large variety of factors, including but not limited to genetic factors, chronic hyperglycemia, physical inactivity, dyslipidemia, pancreatic beta cell dysfunction, chronic inflammation and oxidative and endoplasmic reticulum stress. These events lead to hyperglycemia and complications of diabetes. FFA = free fatty acids; TG = triglyceride; LDL = low-density lipoproteins; VLDL = very-low-density lipoproteins.

The loss of the effect of insulin results in increased glucose formation in the liver, enhanced glycogenolysis, increased lipolysis, and decreased insulin-mediated glucose uptake by skeletal muscle (Figure 3). 

Moreover, the role of obesity as a risk factor for insulin-resistance-induced diabetes is well-documented. Indeed, adipose tissue serves a role not only in fat storage but also as an endocrine organ [28,29]. Several adipocytokines are secreted from adipocytes such as adiponectin, visfatin, leptin, resistin and tumor-necrosis factor α (TNF-α) [30,31,32,33]. A significant reduction in adiponectin and leptin levels are seen in diabetic individuals; this reduction contributes to the impairment of insulin functions [34]. In contrast, the release of inflammatory cytokines such as TNF-α and resistin is increased in obesity and diabetes, which also contributes to insulin resistance by interfering with the insulin signaling pathway [35,36]. Another molecular event that contributes to insulin sensitivity is the reduced level of the incretins glucagon-like peptide 1 (GLP-1) and gastric inhibitory polypeptide (GIP), which are released in the gastrointestinal tract during nutrient absorption after meals [37,38]. The release of incretins augments insulin secretion in healthy individuals; however, this action diminishes in cases of obesity and T2DM. This could be explained by the reduced secretion of incretins or by their deactivation by dipeptidyl peptidase-4 (DPP-4), an enzyme responsible for incretin breakdown. GLP-1 has additional effects, such as suppressing glucagon secretion, stimulating insulin gene expression and the biosynthesis and restoration of glucose competence in glucose-resistant β-cells, hence the use of GLP-1 agonists as hypoglycemic agents [39,40,41,42].

While insulin resistance is the major cause of T2DM, long-standing DM can also impair the insulin-secreting capacity of the pancreas. The reduction in the ability of pancreatic beta cells to produce insulin is due to insulin resistance, among other factors. Chronic hyperglycemia, which induces insulin resistance, was found to reduce the insulin-secreting capacity of pancreatic beta cells by altering metabolic pathways, causing endoplasmic reticulum stress, altering intracellular Ca^2+^ levels and altering the activity of K+− ATP channels [43]. Furthermore, diabetic patients were found to have decreased levels of islet amyloid polypeptide (IAPP), which is another pancreatic peptide co-secreted with insulin [44]. This occurs due to the accumulation of IAPP in the pancreas; IAPP then forms insoluble toxic oligomers that deposit in the β-cells, resulting in its dysfunction.

In addition to the above-mentioned mechanisms, other factors have been reported to contribute to the development of insulin resistance. For example, genetic factors involving mutations in the insulin receptor gene may lead to the development of Type A insulin resistance syndrome [45]. Chronic hyperglycemia has also been implicated in the induction of insulin resistance [46,47] because chronic hyperglycemia can cause oxidative stress and initiate glucotoxicity, which is detrimental to beta cell function. Physical inactivity can also contribute to the development of insulin resistance by increasing the risks of obesity, dyslipidemia, inflammation, ceramide production and oxidative stress and downregulating the Glut-4 and Akt proteins [48]. Inflammation, especially the chronic, low-grade type, has been reported to induce the release of inflammatory mediators such as IL-1β, IL-6, TNF-α, NF-κB, C-reactive protein pro-inflammatory chemokines and cytokines [49]. The dysfunction of pancreatic beta cells may also contribute to the pathogenesis of insulin resistance because a defective beta cell is unable to sense glucose concentration [50]. Dyslipidemia and ectopic fat in insulin-sensitive cells can induce insulin resistance via the increased accumulation of free fatty acids (FFAs), triglycerides (TGs), low-density lipoproteins (LDLs) and very-low-density lipoproteins (VLDLs), which, in turn, cause lipotoxicity [51,52]. Oxidative and endoplasmic reticulum stress (unfolded protein response) play key roles in the pathogenesis of insulin resistance [52,53].

It is worth noting that there is a strong interaction between these factors, indicating that the development of insulin resistance is indeed multifactorial in origin.

## 3. Type 2 DM

Type 2 DM, formerly known as non-insulin-dependent diabetes mellitus (NIDDM), is the most prevalent type of DM [54,55]. The development of this type of DM usually occurs in adulthood, at an age of >40 years, and is characterized by several symptoms such as polyuria, polydipsia, polyphagia and sudden unexplained weight loss. Unlike T1DM, T2DM can be prevented by maintaining a healthy lifestyle that involves regular exercise, smoking cessation, a healthy diet and reversing obesity [8,56,57]. On the other hand, individuals with T2DM are believed to have a higher risk of several complications including stroke, ischemic heart diseases [5], chronic kidney disease [58,59] and increased hospitalization.

Moreover, although it is less commonly seen, poorly controlled T2DM has also been associated with cognitive impairment, symptoms of dementia [60], an increased rate of infections [61] and mild hearing impairment [62]. The time-course of the development of T2DM can take several years, beginning with gradual increments in dysglycemia and moving through prediabetes to overt T2DM [63].

## 4. Management of DM

### 4.1. Lifestyle (Diet and Physical Activity)

Lifestyle modifications have always been considered the cornerstone of DM management and the first step before advancing to pharmacological intervention. These can be defined as the alteration of eating habits and physical activity on a long-term basis in order to reverse obesity, prevent the occurrence of DM and manage DM and other cardiovascular diseases [64]. The effects of lifestyle modifications in reducing the incidence of DM and/or improving glycemic control are well-documented. Obesity is a well-known risk factor for developing DM. One study showed that both men and women with a BMI of 35 kg/m^2^ and greater are 20 times more likely to become diabetic compared to those with a BMI of 18.5–24.9 [65]. A study conducted on individuals with pre-diabetes showed a 20% reduction in the incidence of DM after adopting a healthy lifestyle compared to those with an unhealthy diet and sedentary lifestyle [66,67]. Several studies showed that lifestyle modification and/or pharmacological intervention decreases the occurrence of DM [66,67]. A study showed that combining a healthy diet and regular exercise resulted in a 34–69% reduction in DM over a period of 6 years [68]. In addition, a recent study showed that lifestyle modification in addition to the use of metformin led to a 31–58% reduction in DM over 2 years [69]. Other studies also showed the beneficial role of combining oral antidiabetic agents, such as acarbose and rosiglitazone, with lifestyle modifications; however, the reduction in the incidence of DM was not superior to lifestyle modifications alone [70]. In addition to decreasing the conversion from pre-diabetes to diabetes, adhering to a healthy diet and physical activity can also improve the responsiveness to therapy in diabetic individuals. In fact, diet and exercise improved fasting plasma glucose levels in both obese and non-obese individuals [71]. Indeed, it has been reported that physical exercise activates adenosine-monophosphate-activated protein kinase (AMPK), which plays a role in enhancing glucose uptake in the muscle by stimulating GLUT4 translocation. AMPK also regulates mTOR activities by inhibiting the mammalian/mechanistic target of rapamycin (mTOR). In skeletal muscle, mTOR over-activation can generate insulin resistance through the degradation of IRS-1 via S6K1, leading to a reduced glucose uptake [72].

It is worth noting that lifestyle changes (physical activity and diet) can cause the remission of T2DM. Many trials have shown that this is feasible [73,74].

### 4.2. Diet

One of the most challenging elements in the management of DM is diet and nutrition. While many experts advise diabetic individuals to minimize their intake of carbohydrates and saturated fats, others suggest that eating habits should be individualized, and each diabetic person must be referred to dieticians who are trained to provide diabetes-specific meal plans [75]. There are many diets with proven efficacy in the management of DM, such as the Mediterranean diet, which is high in vegetables, fruits and nuts [76,77], and the Dietary Approaches to Stop Hypertension (DASH) diet [76,78,79]. Although both of these dietary approaches were effective in improving insulin sensitivity and reducing the long-term complications associated with DM, research showed that nutrition distribution for each individual based on current eating patterns, metabolic goals and personal preferences, including financial, traditional and religious factors, is more beneficial in determining the best eating habits [80,81].

Controlling carbohydrate intake has been one of the most important factors for monitoring postprandial hyperglycemia [82,83]. Although many studies showed a 0.2–0.5% reduction in hemoglobin A1C (HbA1c) levels following carbohydrate restriction, the role of a low carbohydrate intake remains unclear, as studies longer than 12 weeks reported no significant improvement in fasting glucose levels or endogenous insulin levels [84].

The literature shows that adjusting daily dietary protein intake provides no evidence in the management of DM in individuals without diabetic kidney disease; however, some studies showed that higher levels of dietary protein may contribute to earlier satiety [78]. In individuals with diabetic kidney disease, careful management of the daily protein intake (0.8 g/kg body weight) is essential in preventing the deterioration of the glomerular filtration rate [85]. 

The recommended daily intake of fat in diabetic individuals is also controversial. Although the National Academy of Medicine (NAM) encourages a fat intake of 20–35% of the total calorie intake [86], studies showed that the type of fat is more critical than the quantity consumed for achieving metabolic goals and that a minimal consumption of saturated fat should be maintained to meet these outcomes [87,88].

### 4.3. Physical Activity

**Figure 4:**
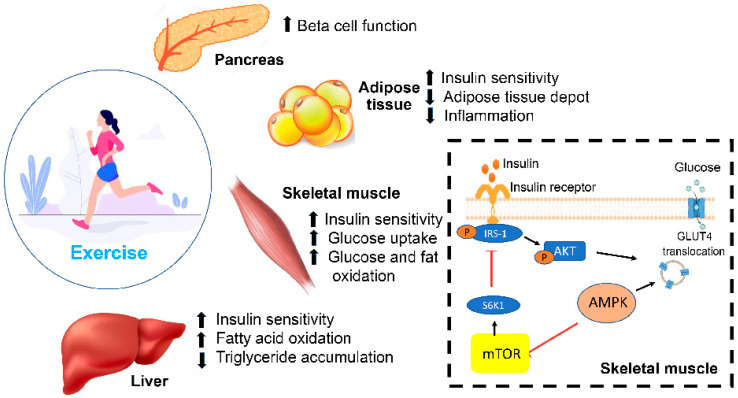
Molecular and physiological effects of physical exercise on glucose metabolism through the pancreas, liver, adipose and skeletal tissues. Physical exercise activates AMPK enhances glucose uptake in the muscle by stimulating GLUT4 translocation.

Physical activity is another essential lifestyle modification that the community in general and diabetic individuals in particular are advised to adapt to. Several types of training are known, including resistance training, aerobic training and high-intensity interval training. While each type of exercise can produce different beneficial outcomes in improving glucose levels and enhancing insulin activity and weight reduction, studies are inconclusive as to the ideal type of exercise for improving metabolic abnormalities [89] (Figure 4).

1. *Resistance exercises*, which involve utilizing free weights and body weight exercises, have been shown to cause a threefold reduction in HbA1c in patients with T2DM when compared to inactive patients [90]. Another study showed that an 8-week weight-training protocol in patients with T2DM improved insulin and glucose responses upon oral glucose tolerance testing [91]. In addition, this type of training caused an increase in the skeletal muscle mass, which is believed to be due to enhanced muscle glycogen storage, leading to increased glucose uptake from the bloodstream. These findings support the benefit of implementing this type of training in a diabetes management plan.
2. *Aerobic training* is another type of exercise that consists of the continuous movement of large muscles, such as in jogging and walking, for at least 30 min per day for 3–7 days weekly, as per the American Diabetes Association (ADA) guidelines [92]. Aerobic training is a well-established tool in improving HbA1c by improving the lipid metabolism and weight loss [93]. One study showed that in 60 adults with T2DM, 6 months of aerobic training caused a significant reduction in HbA1c and fasting insulin levels [94]. Another study showed that aerobic activity in diabetic patients improved glycemic control, insulin sensitivity and oxidative capacity compared to sedentary individuals [95].
3. *Combining both resistance and aerobic exercise* may be the most effective approach to controlling glucose and lipid metabolism in T2DM, as per the current ADA guidelines. Cuff et al. showed that combining both types of exercises led to a significant increase in muscle glucose uptake and insulin sensitivity when compared to aerobic exercises alone [96]. Another distinguished study comparing the effects of both types of exercises alone and their combination in 915 adults showed that individuals utilizing both regimens had a more significant reduction in HbA1c [97].

On the other hand, high-intensity interval training, which consists of four to six repeated intervals of maximal activity interspersed with short periods of rest, recently arose as one of the most practiced exercising modalities [89]. This type of physical activity was found to enhance muscle glycemic control, oxidative capacity and insulin sensitivity in T2DM patients [98,99]. In addition, when comparing the effects of aerobic training to high-intensity interval training, the latter produced a more significant improvement in glucose regulation, insulin resistance and weight reduction [99]. 

## 5. Pharmacotherapy

Several groups of injectable and oral hypoglycemic agents have been discovered for the management of DM (Table 1). Each of these groups contains a number of molecules that share a specific mechanism of action but differ in their pharmacokinetic properties, including the duration of action and/or excretion and metabolism [6,54,100]. As previously mentioned, T2DM is characterized by a myriad of pathophysiological processes, including decreased insulin sensitivity, neurotransmitter receptor dysfunction, decreased pancreatic insulin and increased glucagon secretion, increased gluconeogenesis, increased lipolysis, increased renal glucose reabsorption and a reduction in incretin effects [54,101]. 

Due to the availability of various hypoglycemic agents, each of these pathways can be targeted to control DM and alleviate the symptoms associated with it, such as hyperglycemia, polyuria and fatigue, as well as the long-term complications. In addition, this cocktail of oral and injectable agents can help clinicians in initiating individualized therapies for diabetic patients considering different elements such as efficacy, side effects, costs, comorbidities, weight gain and glucose levels [2,6,121,122,123,124].

Insulin is the mainstay treatment for T1DM and many individuals with T2DM. Although its use in T2DM is unusual for newly diagnosed patients, there are several instances in which the use of insulin is considered, such as severe hyperglycemia, gestational diabetes, the presence of significant weight loss and ketonuria [125,126,127]. It can be administered intravenously, intramuscularly and subcutaneously; however, subcutaneous administration is the predominant route for long-term administration [43,128]. Different preparations of insulin are classified according to their onset and duration of action (Table 2). This includes short-acting, intermediate-acting and long-acting analogs. The different pharmacokinetic properties of these formulations dictate the dosing frequency and the appropriate time for their administration. Long-acting agents such as glargine and levemir are mostly administered at bedtime to cover basal insulin requirements and are associated with a lower incidence of hypoglycemia [129]. Short-acting insulin preparations such as glulisine, aspart and lispro are administered at mealtimes to control post-prandial spikes in glucose levels [130]. Insulin regular is another preparation that is used for emergencies. 

Special training and education are required for patients on insulin therapy due to the complexity of administration and the frequency of dosing. To overcome these difficulties, new insulin preparations are being developed, such as inhaled insulin [131] and oral insulin [132]. In addition, devices such as insulin pumps are used in patients with high HbA1c levels and a history of poor compliance [133]. 

## 6. Current Concepts on Insulin in T2DM 

The HbA1c target is the main goal for the addition of a new drug to the first line of treatment, which is usually metformin and/or basal insulin. Moreover, other factors are also taken into account in selecting an additional anti-diabetic agent, such as the duration of the illness, risk of hypoglycemia, cardiovascular disease and life expectancy. The ADA guidelines recommend that most patients with T2DM should have HbA1c values of less than 7%. Patients with a longer life expectancy, shorter illness duration and no history of cardiovascular disease have an HbA1c target of 6.0–6.5%, while those with a shorter life expectancy, longer duration of illness and history of hypoglycemia have an HbA1c target of 7.5–8.0% [134]. 

The goal of adding insulin to the treatment regimen is to mimic the physiological insulin profile, covering both overnight and postprandial glucose levels. It is recommended by the ADA that long-acting insulin should be incorporated to cover the basal requirement of insulin after the failure of non-insulin agents [134]. In fact, it is believed that intensifying basal insulin therapy in T2DM patients leads to a decrease in glucose toxicity and an improvement in endogenous insulin release from the pancreas [135]. Consistent with this concept, studies showed that short-term insulin therapy in newly diagnosed T2DM patients demonstrated a significant improvement in β-cell function [136,137].

Although insulin has a long duration of action, the postprandial increase in glucose is difficult to control. For this reason, bolus insulin injections may be considered by adding meal-time injections of short-acting insulin, a process called insulin intensification [135]. The advantages and disadvantages of insulin are provided in Table 3. 

## 7. Oral Hypoglycemic Agents

**Figure 5:**
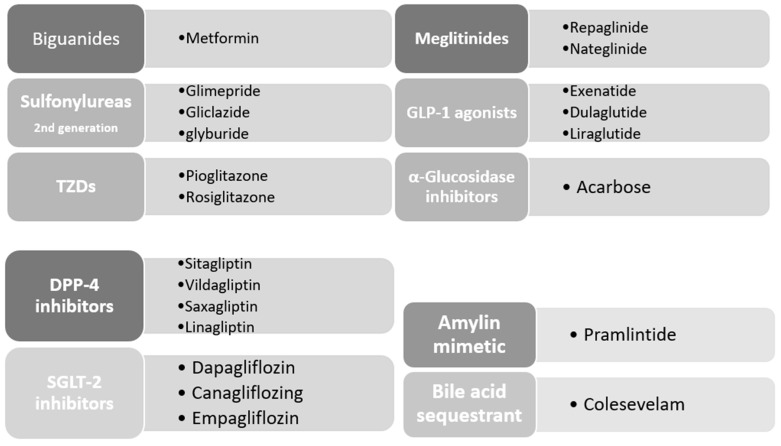
Classes of oral anti-diabetic agents and compounds approved by the American Diabetes Association (ADA).

Many drugs have been approved to lower DM-associated hyperglycemia. These agents include, but are not limited to, biguanides, sulfonylureas, thiazolidinediones, GLP-1 agonists, dipeptidyl peptidase-4 (DPP-4) inhibitors, inhibitors of α-glucosidase, amylin mimetic drugs, bile acid binding resins and sodium–glucose co-transporter (SGLT) inhibitors (Figure 5).

### 7.1. Biguanides

**Figure 6:**
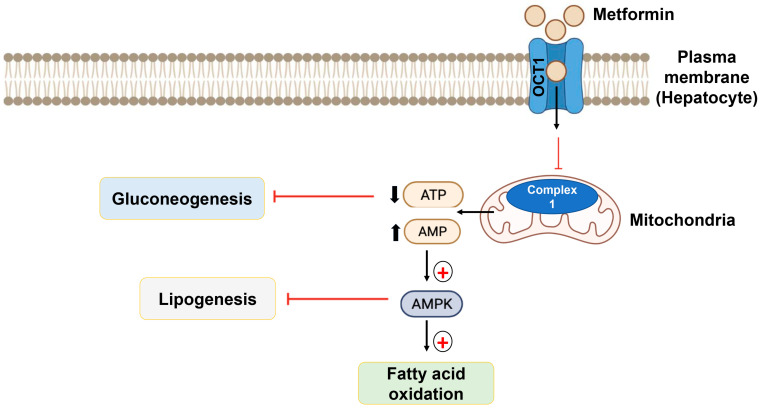
Mechanism of action of metformin. The binding of metformin with organic cation transporter-1 (OCT1) allows the metformin to reach the intracellular region, leading to the activation of AMPK, leading to the oxidation of fatty acid and the inhibition of gluconeogenesis in the liver.

Metformin is the only agent of this group used today. It is the most commonly used agent for T2DM and is accepted as a first-line agent [138]. It operates through the activation of AMP-dependent protein kinase (AMPK), which is activated when cellular energy stores are depleted under normal conditions [139]. The activation of AMPK leads to fatty acid oxidation and inhibits gluconeogenesis in the liver. Moreover, metformin can stimulate glucagon-like peptide-1 (GLP-1) secretion, which improves insulin sensitivity by enhancing the expression of insulin receptors and improving tyrosine kinase activity [102,103,104,105]. Furthermore, metformin can also lower plasma lipid levels and reduce the incidence of cardiovascular disease by acting on peroxisome proliferator-activated receptors (PPAR-αs) [102]. These molecular effects of metformin account for both the hypoglycemic and weight-reducing actions of metformin (Figure 6). 

Metformin can be used as a monotherapy and can also be found in combination with other hypoglycemic agents. Daily dosing varies depending on the formulation (immediate versus extended release), and the length of time on the medication but could range from 500 to 2550 mg daily. The recommended maximum dose of metformin is about 2550 mg daily [140,141].

The side effects caused by metformin mainly involve the gastrointestinal tract, including nausea, vomiting, diarrhea and abdominal discomfort [141]. Therefore, extended-release forms of metformin were developed to reduce the dosing frequency and eventually reduce these side effects. Moreover, the prolonged use of metformin is associated with folic acid and vitamin B12 deficiencies; as a result, monitoring the levels of both vitamins is needed, especially in the elderly [104,142]. Metformin should also be administered with caution and in low doses in patients with heart failure and renal failure as this category of patients have an increased risk of experiencing lactic acidosis, which is considered the most serious side effect of metformin [55]. The advantages and disadvantages of metformin are depicted in Table 4. 

### 7.2. Sulfonylureas

**Figure 7:**
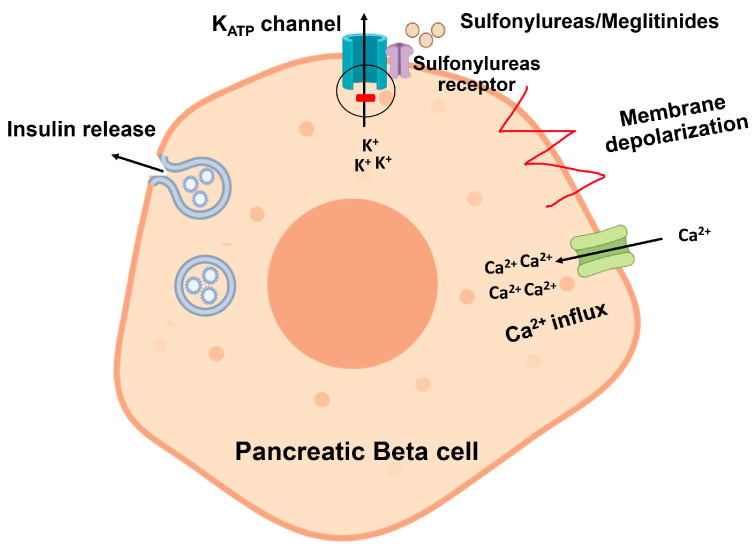
Sulfonylureas bind with the sulfonylurea receptor on the plasma membrane of pancreatic beta cells to block ATP-sensitive potassium channels. This leads to cell depolarization and subsequent increase in calcium-induced insulin release.

Sulfonylureas (SUs) are classified into first and second-generation agents. Due to their frequent dosing and higher risk of hypoglycemia, the first-generation drugs tolbutamide and tolzamide are no longer used clinically [54]. The second-generation agents, such as glibenclamide, gliclazide and glimepiride, are still in use, and some are available in extended-release formulations [142,143]. They exert their effect through the blockade of ATP-sensitive potassium channels found on the pancreatic β-cells, leading to cell depolarization, increasing cellular levels of calcium and enhancing the secretion of insulin, hence the name “insulin secretagogue” (Figure 7). In addition, SUs can also reduce the production of fatty acids and decrease insulin clearance 119. Due to their high efficacy in reducing HbA1c by up to 1–1.5% as and their cost-effectiveness, SUs are considered a second-line therapy and are currently used by 50–80% of diabetic patients worldwide [64,144]. However, prolonged use of these agents reduces their effectiveness. This may be due to progressive β-cell failure or an alteration in the drug’s metabolism.

The major side effect seen with an SU is a weight gain of 1–3 kg. As a result, metformin is provided to patients on SU to reverse weight gain [109,142,145]. Hypoglycemia is also a common side effect, especially with glibenclamide and glimepiride; however, newer agents such as gliclazide have a lower tendency to cause this effect [141]. 

Sulfonylurea-induced hypoglycemia may be caused by decreased renal excretion, hepatic metabolism or displacement from protein-binding sites, which typically occurs in patients with renal/hepatic failure or when co-administered with CYP450 enzyme inhibitors such as aspirin and allopurinol [146]. The advantages and disadvantages of SUs are presented in Table 5. 

### 7.3. Meglitinides

**Figure 8:**
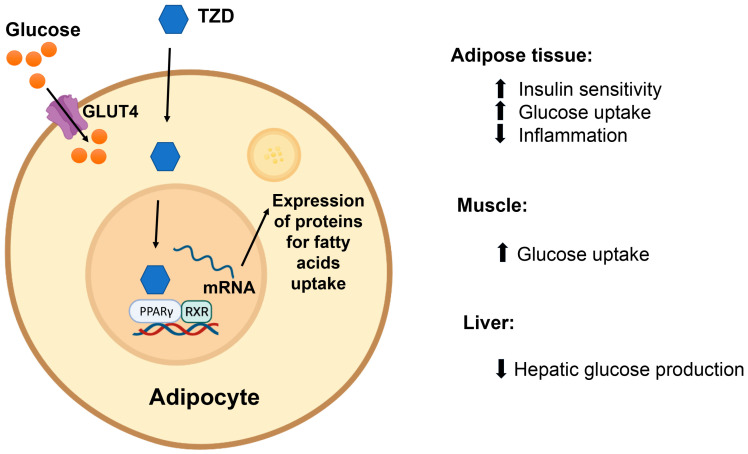
Thiazolidinediones (TZDs) activate PPARγ receptors in adipose tissue to enhance the uptake of circulating fatty acids into adipocytes.

Meglitinides, including repaglinide and nateglinide, belong to another class of insulin secretagogue agents that exert their action by blocking the ATP-sensitive potassium channels in pancreatic β-cells [110] (Figure 8). Unlike SUs, meglitinides have a rapid onset but a short duration of action. These features make them suitable for patients with inconsistent meal times and those who develop rapid postprandial hyperglycemia [100,101,147]. The advantages and disadvantages of meglitinides are shown in Table 6. 

### 7.4. Thiazolidinediones

Two agents from this class are currently used clinically: pioglitazone and rosiglitazone. This pair of agents exerts its hypoglycemic effect by activating PPARγ receptors. PPARγ receptors are expressed primarily in adipose tissue, with lower expression in skeletal muscle, liver, pancreatic β-cells, the central nervous system (CNS) and vascular endothelial cells [55,101,147]. The primary effect of thiazolidinediones (TZDs) is believed to be through the activation of PPARγ receptors in adipose tissue, which promotes the uptake of circulating fatty acids into fat cells, thereby increasing insulin sensitivity (Figure 8). 

Moreover, activation of this receptor in skeletal muscle and the liver also contribute to TZD action as they increase glucose uptake and reduce glucose production in both organs, respectively. TZD can cause a 0.5–1.4% reduction in HbA1c, and clinical trials showed a 10–15% reduction in plasma triglyceride levels [106,107]. In fact, this effect on the lipid profile is believed to be mediated through another isoform of PPAR receptors present in the liver, heart and skeletal muscles [148]. Furthermore, TZDs were also found to reduce the levels of inflammatory cytokines, such as tumor necrosis factor alpha, improving the function of pancreatic β-cells and increasing the levels of adiponectin, both of which are believed to contribute to their insulin-sensitizing effects [55]. 

The common side effects of TZDs include weight gain and edema. These are believed to occur because of the activation of PPARγ receptors in the CNS, which increases food intake [116]. Studies showed that TZDs can also increase the risk of bone fracture in women and caused a reduction in transaminases; therefore, they should be avoided in patients with liver disease. Rosiglitzone has also been associated with an increase in myocardial infarction incidence [149]. The advantages and disadvantages of TZDs are provided in Table 7. 

### 7.5. Glucagon-like Peptide-1 (GLP-1) Agonists 

**Figure 9:**
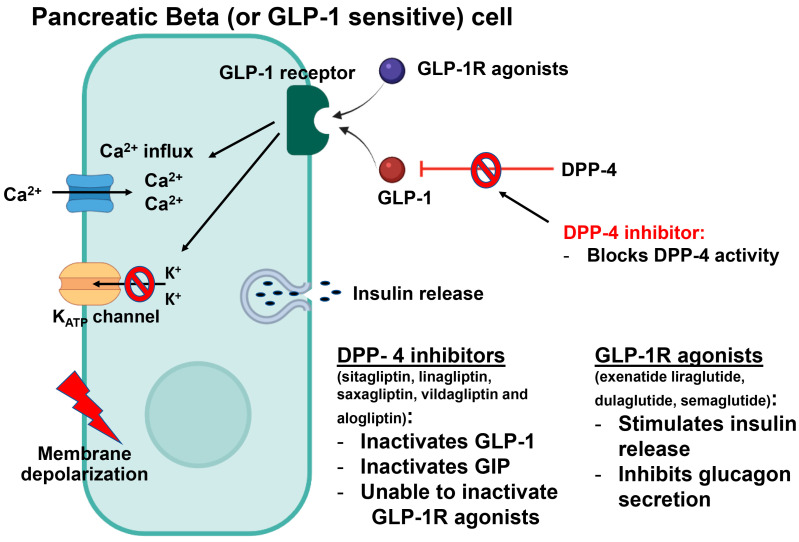
GLP-1 binds to GLP-1R and activates a cascade of intracellular events, leading to insulin release and the inhibition of glucagon production.

Glucagon-like peptide-1 (GLP-1) is an incretin secreted from the distal ileum in response to nutrients such as proteins and carbohydrates [150,151,152]. Following its release, GLP-1 binds to its receptor, GLP-1R, which is expressed on the pancreatic β-cells, thereby activating a cascade of intracellular events that increases the release of insulin, inhibits the release of glucagon, reduces food intake by causing satiety, delays food emptying and normalizes both postprandial and fasting insulin secretion [111] (Figure 9).

Patients with DM were found to have a significant reduction in the levels of GLP-1, which is believed to occur due to a reduction in the expression of GLP-1 receptors in the pancreas [112,113] or an enhancement of DPP-4 activity [113]. Due to its potent insulinotropic effects, restoring the activity of GLP-1 arose as a potential target for researchers and pharmaceutical companies. As a result, several GLP-1 agonists have been developed and used clinically in the management of T2DM [119,153,154], including exenatide, liraglutide and dulaglutide [55,141]. These molecules are administered subcutaneously and have various pharmacokinetic properties accounting for the differences in dosing. The majority of side effects associated with the administration of these compounds involve the GI tract, and this includes diarrhea, nausea and vomiting. In addition, some patients reported abscesses, cellulitis formation and even tissue necrosis at the site of injection [153,155]. The risk for hypoglycemia is low unless they are used in combination with insulin or sulfonylureas. The advantages and disadvantages of incretins are provided in Table 8. 

It is worth noting, however, that the stimulation of GLP-1R has many other effects outside of the pancreatic and the gastrointestinal systems. These effects range from neuroprotective action and nerve growth promotion to the ability to improve cardiovascular function [156].

### 7.6. Dipeptidyl Peptidase-4 (DPP-4) Inhibitors 

DPP-4 is a serine protease that is expressed on endothelial cells and T-lymphocytes and in a free-circulating form. Its main function is the inactivation of the glucagon-like peptide-1 (GLP-1) and gastric inhibitory peptide (GIP) produced in the intestines [42,157]. These two hormones are known as incretins, and they play an essential metabolic role in augmenting the secretion of insulin, inhibiting glucagon secretion and reducing the absorption of nutrients [119,120]. By inhibiting this enzyme, DPP-4 inhibitors are considered oral hypoglycemic agents that are used widely in the management of DM (Figure 9).

Several DPP-4 inhibitors are available nowadays, such as sitagliptin, linagliptin, saxagliptin, vildagliptin and alogliptin. They can be used alone or in combination, and studies have shown a 0.48–0.6% reduction in HbA1c and >95% decrease in the activity of DPP-4 for 12 h [42,119]. 

Unlike the previously mentioned antidiabetic agents, DPP-4 inhibitors have no effect on insulin sensitivity or secretion; as a result, weight gain is not an adverse effect of gliptins [145,154,158]. Sitagliptin, saxagliptin and vildagliptin are excreted renally; therefore, dose adjustment is required for diabetic patients with moderate to severe renal disease. Linagliptin, on the other hand, is excreted by the enterohepatic system, so it can be used as the agent of choice in renal impairment [159].

Although they cause minimal to no weight gain and have a low incidence of hypoglycemia, DPP-4 inhibitors are associated with other side effects such as nasopharyngitis, upper respiratory tract infections and headaches. In addition, these agents were found to cause pancreatitis and hepatic dysfunction after prolonged use [160]. The advantages and disadvantages of incretins are depicted in Table 9. 

### 7.7. α-Glucosidase Inhibitors

Acarbose and miglitol are two agents of this class that are available and used clinically. α-glucosidase is an enzyme responsible for the breakdown of oligosaccharides into monosaccharides, and inhibiting it causes a reduction in intestinal glucose absorption by delaying the digestion of carbohydrates [2,5,59,117,118]. Moreover, these compounds were also reported to augment the release of GLP-1, which also contributes to their HbA1c-lowering activity (0.5–0.8%) [101,141]. The major side effects associated with this class are flatulence, diarrhea and abdominal pain [141]. The advantages and disadvantages of α-glucosidase inhibitors are provided in Table 10. 

### 7.8. Amylin Mimetic

**Figure 10:**
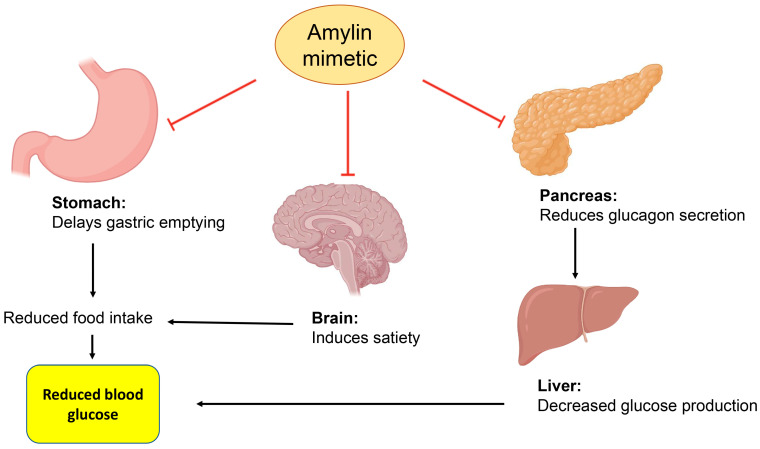
Amylin delays gastric emptying, increases satiety, and reduces glucagon secretion.

Amylin is a pancreatic hormone co-secreted with insulin from β-cells in the pancreas, and it acts by reducing the secretion of glucagon, delaying gastric emptying, and inducing satiety [114,115,116]. (Figure 10). Pramlintide is the only available amylin mimetic approved for use by the Food and Drug Administration. It is administered subcutaneously, and it is used in both T1DM and T2DM [161,162]. The advantages and disadvantages of amylin are provided in Table 11.

### 7.9. Bile Acid Binding Resins

**Figure 11:**
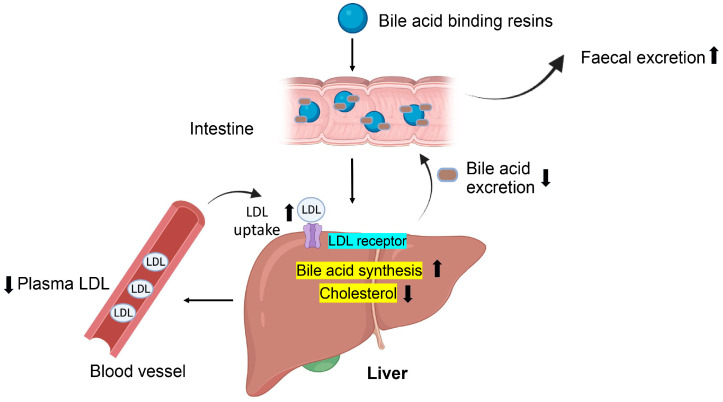
Bile acid binding resins stimulate LDL-receptors on hepatocytes to enhance the uptake of LDL proteins from blood circulation.

Colesevelam is the only agent in this class of hypoglycemic agents. Although it does not have a direct effect on insulin secretion and/or sensitivity, the glucose-lowering mechanism of bile acid sequestrants is mostly unknown [163,164]. It is known, however, that colesevelam can reverse dyslipidemia, which is recognized as an exacerbating factor in T2DM. Current data suggest that colesevelam alone can produce a 0.5% reduction in HbA1c and a 13–17% reduction in low-density lipoproteins (LDL) [165]. A lack of systemic side effects makes this a good adjunct medication for managing T2DM (Figure 11). 

### 7.10. Sodium–Glucose Co-Transporter (SGLT) Inhibitors

**Figure 12:**
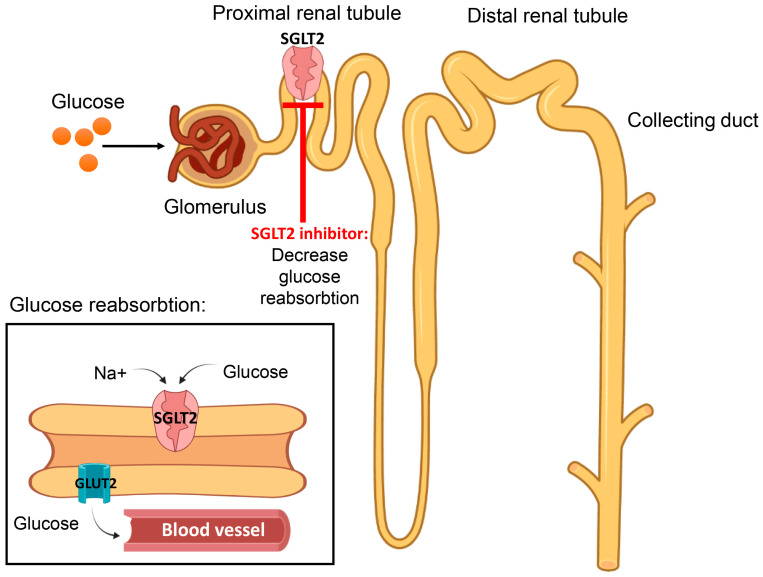
Sodium–glucose co-transporter (SGLT) inhibitors prevent the reabsorption of glucose from the proximal convoluted tubule, thereby reducing blood glucose level by increasing excretion.

This is a newer class of antidiabetics that was introduced clinically in 2013, with canagliflozin being approved by the Food and Drug Administration (FDA) [166,167]. These molecules exert their action on the renal sodium–glucose co-transporter-2 (SGLT2) molecule, which is responsible for glucose reabsorption in the proximal renal tubules [108] (Figure 12).

This novel agent stimulates glucose excretion and has also been shown to have weight loss effects with minimal hypoglycemia [168,169]. In fact, canagliflozin was reported to cause a significant reduction in HbA1c of 0.77–1.03% [170], and dapagliflozin produced similar results after both short- and long-term treatments [171]. Another type of SGLT exists which is found in the intestines and the proximal convoluted tubules of the kidneys [172,173]. Although SGLT2 is responsible for the reabsorption of 90% of glucose filtered via glomeruli, diabetic patients with declining renal function may respond less to SGLT2 inhibitors, making SGLT1 inhibitors a better option for treatment [173]. Furthermore, dual SGLT1 and SGLT2 inhibitors such as sotagliflozin and licogliflozin are currently being investigated and are expected to have an agonistic hyoyglycemic effect while enhancing GLP-1 release from the intestines [173]. Currently, three types of SGLT2 inhibitors have been approved for use in the United States, including dapagliflozin, empagliflozin and the prototype SGLT2 inhibitor canagliflozin [169], while sotagliflozin is under investigation. In general, these agents have a good pharmacokinetic profile, including excellent oral bioavailability, a long half-life and limited renal excretion; however, they increase the risk for genital and urinary tract infections and orthostatic hypotension [159]. The advantages and disadvantages of SGLT2 inhibitors are provided in Table 12.

## 8. Effectiveness of Different Classes of Anti-Diabetic Drugs on HbA1c

Table 13 presents the degree of HbA1c reduction by class of drug, which is an important measure for long-term glycemic control. The reports from the literature, as described in previous sections, show that metformin and second-generation sulfonylureas are the most effective agents for the reduction of HbA1c.

## 9. Anti-Diabetic Drugs That Have Been Suspended

Despite their significant glucose-lowering activity, several agents have been discontinued by the Food and Drug administration (FDA) due to safety concerns. Troglitazone was the first TZD to be approved, but it was withdrawn due to the emergence of severe liver toxicity that resulted in 90 deaths [174]. Another agent that was put under heavy restrictions by the FDA is rosiglitazone. Rosiglitazone was associated with increased risk of heart conditions, including heart failure, stroke and death [175]. However, the restrictions on rosiglitazone were lifted in 2013 after a review of clinical data. First-generation Sus, including acetohexamide, chlorpropamide, tolazamide and tolbutamide, which were released in the 1960s, were replaced by second-generation SUs due to several side effects such as a disulfiram-like reaction and hepatotoxicity [176]. Moreover, two insulin combinations, ryzodeg and novolog, were recently withdrawn by the FDA due to concerns about cardiovascular side effects [177].

## 10. New Directions for the Prevention and Management of Diabetes Mellitus

Moreover, as prevention is known to be better than a cure, we can expect that genetic investigations can be utilized to screen individuals who are at risk of developing the disease and to detect genes responsible for the development of T2DM. In fact, genetic testing is already used in the diagnosis of MODY [178] and has been proposed for T2DM [179]. Using the genetic testing approaches [180] will not only aid in reducing the incidence of the illness but will also be crucial in the pharmacogenomic aspect of therapy via selecting the ideal treatment for each individual, which can optimize treatment outcomes. In addition to therapeutic advances, it is important to capitalize on the potential of lifestyle modifications in the management of T2DM. 

## 11. Conclusions

Type 2 diabetes mellitus (T2DM) is a complex metabolic disorder that affects various organ systems and is multi-factorial in origin. Addressing the main issue of insulin resistance reduces the incidence of long-term complications of T2DM. Its management involves lifestyle modifications and pharmacotherapy with traditional and novel antidiabetic agents. Despite the variety of pharmacological agents currently available for the management of T2DM, research to discover novel targets that may broaden and individualize treatment options is ongoing. With our review, we hope to provide the latest updates on current and novel treatment regimens for T2DM, which may guide healthcare providers in managing this chronic disease. 

## Figures and Tables

**Table 1:** Anti-diabetic agents used clinically, their target organs and their mechanisms of action.

Drugs	Organ Targeted	Mechanism	References
TZD and biguanides	Adipose tissue Skeletal muscle	↓ Insulin resistance	[102,103,104,105,106,107]
TZD and biguanides	Liver	↓ Gluconeogenesis	[55]
SGLT2 inhibitors	Kidney	Glucose elimination in urine	[108]
SU and meglitinides	Pancreas	Insulin secretagogues	[109,110]
GLP-1R agonists	Pancreas	Improve response to glucose	[111,112,113]
Pramlintide	Pancreas	↓ Glucagon secretion	[114,115,116]
Pramlintide	Stomach	Delays gastric emptying	[115]
α-glucosidase inhibitors	Small intestine	Slows absorption of starch	[117,118]
DPP-4 inhibitors	Plasma	↓ Incretin breakdown	[119,120]

TZD = thiazolidinediones; SGLT2 = sodium–glucose transporter-2; SU = sulfonylureas. GLP-1R = glucagon-like peptide-1; DPP-4 = dipeptidyl peptidase 4.

**Table 2:** Types of insulin preparations and their pharmacokinetic profile (modified after Kaufman, 2003 [129]).

Insulin Type	Onset of Action (h)	Peak of Action (h)	Duration of Action (h)	Maximal Duration (h)
**Rapid-acting**				
Lispro	¼ to ½	1 to 2	3 to 5	4 to 6
Aspart	¼ to ½	1 to 2	3 to 6	5 to 8
Glulisine	0.25 to 0.5	0.5 to 1	3 to 4	4
**Short-acting**				
Regular	½ to 1	2 to 4	3 to 6	6 to 8
**Intermediate-acting**				
NPH human	2 to 4	8 to 12	12 to 20	14 to 22
**Long-acting**				
Glargine	1 to 2	None	19 to 24	24
Detemir	3 to 4	6 to 8	20 to 24	24
Degludec	1	9	24 to 42	42
**Insulin combinations**				
Protamine/Lispro	0.25 to 0.4	0.5 to 3	14 to 24	24
Protamine/Aspart	0.1 to 0.2	1 to 4	18 to 24	24

**Table 3:** Advantages and disadvantages of insulin in T2DM.

Daily Dosage	Advantages	Side Effects	Contraindications
Initial dose: 0.5–1 unit/kg per day Maintenance dose: individualized to achieve blood glucose levels of 80–140 mg/dL	Fewer episodes of hyperglycemia	Weight gain Hypoglycemia Injection site reactions Lipodystrophy	Hypersensitivity Liver disease Kidney disease

**Table 4:** Advantages and disadvantages of metformin.

Daily Dosage	Advantages	Side Effects	Contraindications
500–2550 mg (depending on immediate vs. extended-release formulations)	Weight loss Inexpensive	Diarrhea Vomiting Dyspepsia Flatulence Metallic taste Lactic acidosis	Renal disease Heart failure Liver disease Hypoxic pulmonary disease

**Table 5:** Advantages and disadvantages of sulfonylureas.

Daily Dosage	Advantages	Side Effects	Contraindications
Glibenclamide (1.25–20 mg) Glimepiride (1–8 mg) Gliclazide (30–120 mg)	Rapid effectiveness	Weight gain Hypoglycemia GI distress Dizziness	Pregnancy Ketoacidosis

**Table 6:** Advantages and disadvantages of meglitinides.

Daily Dosage	Advantages	Side Effects	Contraindications
Repaglinide (0.5–4 mg) Nateglinide (60–120 mg)	Ideal for postprandial glucose increase Ideal for patients with irregular meal schedule	Weight gain Hypoglycemia	Pregnancy Hypersensitivity Co-administration of gemfibrozil with repaglinide

**Table 7:** Advantages and disadvantages of thiazolidinediones.

Daily Dosage	Advantages	Side Effects	Contraindications
Pioglitazone (15–45 mg) Rosiglitazone (4–8 mg)	Improve lipid metabolism	Fluid retention Weight gain Bone loss	Active liver disease Patients with heart failure (Class III; IV)

**Table 8:** Advantages and disadvantages of incretins.

Weekly Dosage	Advantages	Side Effects	Contraindications
Exenatide (2 mg) Liraglutide (0.6–3.0 mg) Dulaglutide (0.75–1.5 mg) Semaglutide (0.25–0.5 mg)	Low hypoglycemia Weight loss Lowering blood pressure and cardiovascular disease	Nausea	Pancreatitis Renal impairment

**Table 9:** Advantages and disadvantages of Dipeptidyl peptidase-4 (DPP-4) inhibitors.

Daily Dosage	Advantages	Side Effects	Contraindications
Sitagliptin (50–100 mg) Saxagliptin (2.5–5 mg) Vildagliptin (50–100 mg) Linagliptin (5 mg)	Weight loss	Hypoglycemia Pancreatitis GI distress Flu-like symptoms Joint pain	Pregnancy Pancreatitis Heart failure Angioedema

**Table 10:** Advantages and disadvantages of α-Glucosidase inhibitors.

Daily Dosage.	Advantages	Side Effects	Contraindications
Acarbose (25–300 mg)	Minimal risk of hypoglycemia	GI distress	Liver cirrhosis Colonic ulceration Inflammatory bowel disease

**Table 11:** Advantages and disadvantages of Amylin mimetics.

Daily Dosage.	Advantages	Side Effects	Contraindications
Pramlinitide (30–60 µg)	Weight loss	Nausea	Gastroparesis Asymptomatic hypoglycemia

**Table 12:** Advantages and disadvantages of SGLT inhibitors.

Daily Dosage	Advantages	Side Effects	Contra-Indications
Dapagliflozin (5–10 mg) Empagliflozin (10–25 mg) Canagliflozin (100–300 mg) Sotagliflozin (200–400 mg)	Increase GLP-1 release Weight loss Reduce blood pressure Reduce triglycerides	GI distress Hypoglycemia Urinary tract infections	Dialysis Renal impairment

**Table 13:** Effect of different classes of anti-diabetic drugs on HbA1c.

Class of Drug	Expected Reduction in HbA1c	Contraindications
Biguanides (Metformin)	1.0–2.0%	Renal dysfunction Hepatic dysfunction Congestive heart failure
TZD	0.5–1.4%	Class III and IV heart failure
Sulfonylureas	1.0–2.0%	Pregnancy Ketoacidosis
DPP-4 inhibitors	0.5–0.8%	Pancreatitis
SGLT-2 inhibitors	0.7–1.0%	Severe renal impairment End stage renal failure
Meglitinides	0.5–1.5%	Co-administration with gemfibrozil
α-Glucosidase inhibitors	0.5–0.8%	Liver cirrhosis Inflammatory bowel disease Intestinal obstruction
GLP-1 agonists	0.5–1.0%	History of medullary thyroid carcinoma
Amylin mimetic	0.5–1.0%	Gastroparesis Hypoglycemia
Bile acid sequestrant	0.5–0.9%	Elevated triglycerides
